# The universal stem cell

**DOI:** 10.1038/s41375-022-01715-w

**Published:** 2022-10-28

**Authors:** Peter J. Quesenberry, Sicheng Wen, Laura R. Goldberg, Mark S. Dooner

**Affiliations:** 1 Division of Hematology/Oncology, Brown University, Rhode Island Hospital, Providence, RI 02903 USA; 2 Division of Hematology, Brigham and Women’s Hospital, Boston, MA 02115 USA

**Keywords:** Haematopoietic stem cells, Stem-cell research

## Abstract

Current dogma is that there exists a hematopoietic pluripotent stem cell, resident in the marrow, which is quiescent, but with tremendous proliferative and differentiative potential. Furthermore, the hematopoietic system is essentially hierarchical with progressive differentiation from the pluripotent stem cells to different classes of hematopoietic cells. However, results summarized here indicate that the marrow pluripotent hematopoietic stem cell is actively cycling and thus continually changing phenotype. As it progresses through cell cycle differentiation potential changes as illustrated by sequential changes in surface expression of B220 and GR-1 epitopes. Further data indicated that the potential of purified hematopoietic stem cells extends to multiple other non-hematopoietic cells. It appears that marrow stem cells will give rise to epithelial pulmonary cells at certain points in cell cycle. Thus, it appears that the marrow “hematopoietic” stem cell is also a stem cell for other non-hematopoietic tissues. These observations give rise to the concept of a universal stem cell. The marrow stem cell is not limited to hematopoiesis and its differentiation potential continually changes as it transits cell cycle. Thus, there is a universal stem cell in the marrow which alters its differentiation potential as it progresses through cell cycle. This potential is expressed when it resides in tissues compatible with its differentiation potential, at a particular point in cell cycle transit, or when it interacts with vesicles from that tissue.

## Introduction

The Hematopoietic stem cell (HSC) has been exhaustively defined as a dormant non-cycling lineage negative cell expressing c-kit, Sca-1 and CD150, with the capacity to differentiate into hematopoietic end cells and to self-renew. HSCs have been shown to be influenced by tissue microenvironments and circulating cytokines. The cell has been most thoroughly characterized in murine transplant studies by determining the capacity to renew multilineage hematopoiesis in lethally irradiated mice. It has been isolated by removal of cells by fluorescent activated cell sorting utilizing differentiation lineage defining antibodies followed be selection with stem cell defining antibodies most commonly anti-Sca-1, anti c-Kit and anti CD150. The potential impact of the cell separation is, of course, important. Antibody ligation of stem cells could of course be stimulatory or inhibitory and needs to be worked out. This should be an aspect of future studies. In a similar fashion the cellular effects of dye exclusion approaches need to be evaluated. An additional consideration is the potential effect of helper cells on stem cell activity. There is also a large literature on helper T cells and stem cells which can be regarded as a separate issue with regard to this review. A review in 2015 by Goodell and colleagues [1] noted: “It is striking that more than 100 years after the term “stem cell” was coined, we are again debating the interrelationship among different cell types in various tissues”. They go on to state “A key remaining question is whether there is one “uber” stem cell in each of these tissues”. This goes to the heart of our new model of stem cell existence and regulation. In their review they outline three possible models; a traditional model, a consortium model with a pool of stem cells with slightly different properties and a speculative model in which stem cells are seen as rare reserve cells that occasionally generate lineage restricted progenitors [1].

We propose that there is, in fact, a single cell which serves as a stem cell for all tissues. The phenotype of this cell is determined by its cell cycle state and by the tissue in which it resides. Here we outline our data and reasoning for this model. Four major considerations are reviewed (Table 1): (1) alterations of cellular phenotype with cell cycle passage of the stem cell at baseline state, (2) differentiation potential of the stem cell at different points in cell cycle (3) microenvironmental influences and stem cell plasticity and (4) stem cell heterogeneity. All of these closely interrelated considerations point to the existence of a cycling universal stem cell for all tissues.

**Table 1:** Key considerations for the universal stem cell model.

1. Cell cycle status at baseline and changes of phenotype with cycle transit
2. Differentiation at different points in stem cell cycle
3. Microenvironment and stem cell plasticity—marrow stem cells differentiating into non-hematopoietic cells
4. Heterogeneity of described stem cells

## Cell cycle status at baseline and changes of phenotype with cycle transit

We had proposed a continuum model for hematopoiesis as early as 2002 [2, 3]. In continuing studies, marrow stem cells were studied at different points in a cytokine induced cell cycle transit. The cells studied included whole unseparated marrow, lineage negative rhodamine low Hoechst low (LRH) separated marrow cells and lineage negative Sca-1 positive cells stimulated to transit cell cycle with either interleukin 3 (IL-3), IL-6, IL-11 and steel factor, or thrombopoietin, Flt3 ligand and steel factor. Hematopoietic stem cells were observed to reversibly alter phenotype at different points in cell cycle. These changes were seen in short and long term engraftment into lethally irradiated mice [4], in homing to marrow [5], in progenitor to stem cell ratios [6], in expression of adhesion receptors [7, 8] and general gene expression [9], and in differentiation into megakaryocytes and granulocytes [10]. These studies were of cells progressing through a cytokine stimulated cell cycle transit. It appeared that the phenotype of primitive long-term re-populating murine stem cells changed dramatically as they progressed through cell cycle. Our data indicated that hematopoietic stem cells were on a continuum of change, rather than in the classically described stem/progenitor hierarchy. Our data was heavily dependent on marrow populations stimulated with cytokines in in vitro cell culture. Thus, the elegant work of Passegue et al. [11] was important to us. These investigators evaluated lineage negative, c-Kit^+^ Sca-1 + Thy1.1^int^Flk2^int^ cells, long-term hematopoietic reconstituting cells, separated by Hoechst 33422 P and Pyronin Y staining for DNA/RNA content and then evaluated them at different points in cell cycle for long term engraftment into lethally irradiated mice. The following fractions were studied; G0 (very low Pyronin 2n DNA), G1 (increasing pyronin) and S/G2/M (increasing DNA content by Hoechst staining). Engraftment was only seen in the G0 population. This suggested that our data on cycle dependent phenotype changes might not be applicable to baseline stable hematopoietic stem cells and that the data supporting a continuum stem cell model might relate to in vitro culture artifacts. We proceeded to evaluate purified stem cells separated as per Passegue et al. [11]. Essentially, we confirmed their results, showing long-term engraftment almost totally in the G0 group. However, while considering these data we noted that no one had ever evaluated the cell cycle status of unseparated whole murine marrow long-term repopulating stem cells. We have been intrigued with the work of Kahneman and Tversky [12] and felt that representative and availability heuristics might apply here. Much data supported the purification approach, the obviousness of the hierarchical stem cell model with differentiation to end cells, thus, diminishing pursuit of alternative possibilities. Thus, we proceeded to evaluate the cell cycle status of long-term re-populating multilineage stem cells in unseparated whole marrow cells. Cells were separated into G0, G1 and S/G2/M or G0/G1 and S/G2/M, cells by Hoechst alone or Hoechst 33342/PyroninY staining as described by Passegue et al. [11], evaluating their long-term re-populating capacity in lethally irradiated C57BL/6 J hosts. We determined blood and marrow chimerism out to 12 months [13]. There was consistently 50% or more engraftment within the S/G2/M fraction (3 separate experiments and 10–17 mice per time point, *p* > 0.05 by Wilcoxon rank-sum analysis), a sharp contrast to results seen with purified marrow stem cells. This was an instantaneous look at cell cycle. Finally, in one experiment serial transplant of S/G2/M marrow 1 year after the initial transplantation was superior of that seen with G0/G1 marrow.

These were controversial data, so we sought to confirm these results using different approaches. In addition, there is always some overlap of cell populations with flow cytometry, so we elected to evaluate the cell cycle status of purified stem cells and stem cells in unfractionated marrow using tritiated thymidine suicide to evaluate stem cell cycle status. In this approach high specific activity ^3^H-thymidine incorporates into growing DNA strands and kills cells in S-phase. Cell mixture experiments assured against innocent bystander effects, although the path length of this beta emitter also indicates that there would be no innocent bystander effects. WBM was incubated in vitro for 30 min with ^3^H-thymidine prior to transplantation. Controls included non-radiolabeled thymidine and un-manipulated WBM. There was a 65–80% reduction in engraftment at 1, 3, 6 and 12–14 months into lethally irradiated host mice in the group of WBM treated with tritiated thymidine (25–28 mice per time point, 4 experiments, *P* < 0.001) [13]. Thus, experiments using thymidine suicide showed that long-term repopulating stem cells were cycling.

In addition, we characterized the in vivo flux of LT-HSCs (Lineage^−^/c-Kit^+^/Sca-1^+^/Flk-2^−^) through cell cycle utilizing in vivo administration of BrdU, both intraperitoneally and orally. At 12, 24 and 48 h after initiation of BrdU we observed that 39%, 65%, and 72% of the LT-HSC were labeled and 31%, 58% and 67% of these cells were in G0/G1. Thus, they had passed through cell cycle during these time intervals. This is another indication, along with the cell cycle separated marrow and tritiated thymidine experiments, that LT-HSCs from marrow are actively cycling.

**Fig. 1:**
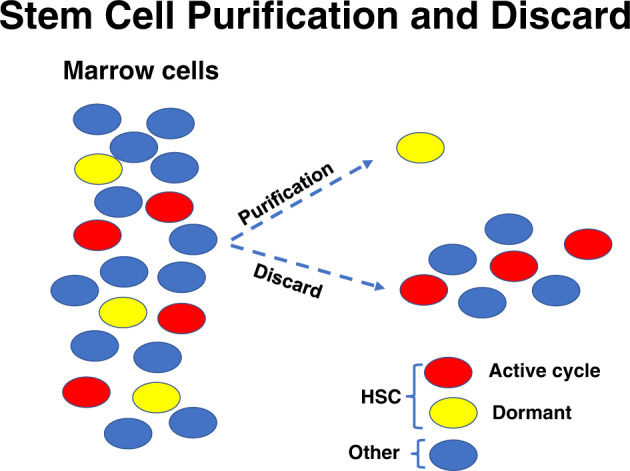
Standard stem cell purification. Yellow circles are purified non-cycling marrow stem cells, i.e., Lineage negative c-Kit + , CD150 + and Sca-1+ cells, red circles are cycling c-Kit+CD150 + and Sca-1+ stem cells. Blue circles are other lineage positive marrow cells.

These cycling cells are removed with standard stem cell purification approaches (Fig. 1). This is further demonstrated by experiments showing significant re-populating stem cell content in the lineage positive population separated from whole marrow [13]. There was an 84–99% reduction in engraftment in the tritiated thymidine exposed population (*P* < 0.02 by Wilcoxon rank sum) showing that these cells were proliferating.

These data definitively show that long-term multilineage marrow stem cells are in active cell cycle and that these cells are discarded with the lineage depletion of marrow cells during standard stem cell purification approaches.

## Hematopoietic differentiation potential

**Fig. 2:**
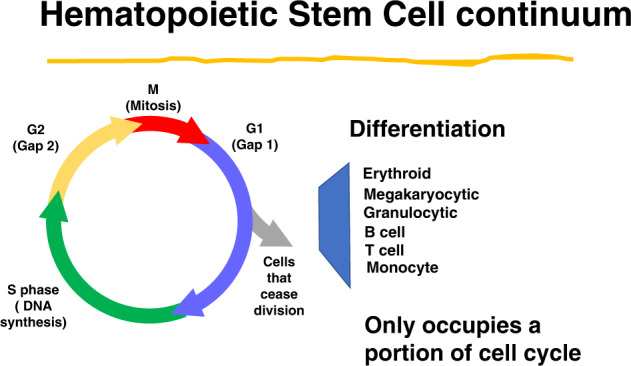
The continuum isolated to hematopoietic cells. Large circle is cell cycle transit. Differentiation to different hematopoietic fates only occurs during a specific phase of cell cycle.

A critical feature of marrow stem cells is their capacity for differentiation. This is also tied to their cell cycle status. Our early studies on the differentiation of LRH stem cells indicated cycle related “hotspots” for both murine granulocytes and megakaryocytes. LRH are separated based on their quiescence, and they are highly synchronized in cell cycle by the purification. These are perhaps one of the most purified hematopoietic stem cells [14–18]. In single cell transplants one in three to one in four will engraft long term. They are tightly synchronized when cultured in thrombopoietin (TPO), FlT-3 ligand and steel factor as determined by propidium iodide (PI) analysis, cell-doubling time and tritiated thymidine uptake [6, 18, 19]. We evaluated differentiation of these stem cells at different times in cytokine (steel factor, Flt-3 ligand and TPO) stimulated cell cycle transit [10]. We ascertained their differentiation in response to G-CSF, GM-CSF and steel factor after 14 days in culture and found marked increases in megakaryocytes at 32 h of cycle transit (G1/early S phase). In a total of seven experiments, the mean increase at 32 h was significantly different from 0 h (*p* = 0.028). Megakaryocytes were identified by Wright-Giemsa, acetyl cholinesterase and Von Willebrand factor staining and expression of CD41. Levels returned to baseline at latter times in cycle transit. In a similar fashion we observed a “hot spot” for differentiation into metamyelocytes, bands, and polymorphonuclear granulocytes (non-proliferative granulocytes) at mid S-phase (*p* = 0.043). Thus, there exists points on a cell cycle transit where cytokines induce specific hematopoietic lineages. These data were restricted to hematopoietic lineages, but there is abundant evidence for marrow stem cell differentiation into non-hematopoietic tissues; so-called “stem cell plasticity” (see below). We established that lineage positive marrow cells were replete with stem cells and these were discarded with the stem cell purification [13]. This indicated that stem cells express differentiation antigens. We proceeded to separate populations of marrow cells expressing single specific differentiation antigens. When B220 or GR-1 positive marrow cells were separated, abundant numbers of long-term repopulation stem cells were found. A second cell sort was carried out to improve “purity” of the population. In these experiments the long-term re-populating stem cell content was no longer present in the B220 or GR-1 double sorted cells [20]. However there now appeared a separate “minor” population of B220 or GR-1 negative cells, which contained the stem cells. This population was positive for other differentiation epitopes and the isolated stem cells were c-Kit, Sca-1, CD150 positive and actively cycling by tritiated thymidine suicide experiments. We interpret these data as indicating that the marrow stem cells express differentiation antigens on their cell surface, in this case B220 or Gr-1, and that this expression is tied to phase of cell cycle. Since the time intervals between the first and second stem cell assays (with these sequential cell-sorts) was approximately 2–3 h it would appear that the stem cells had lost expression of B220 or GR-1 during these short time intervals. We hypothesize that multiple other differentiation antigens are expressed by these cells, as indicted by the stem cell plasticity studies, and that the expression of these varies with cell cycle phase. The final fate of the stem cell at a specific cell cycle phase will then be determined by its tissue residence. For example, a marrow stem cell expressing pulmonary epithelial antigens will preferentially form epithelial cells in lung, but not in the marrow, while if it is expressing hematopoietic differentiation antigens at a different point in cell cycle progression it will form hematopoietic end cells, preferentially in bone marrow. The type of end cell here will vary depending on point in cycle passage (Fig. 2). The alteration of gene expression with passage through the first cycle from dormancy is consistent with this postulate [21, 22].

## Microenvironment and stem cell plasticity—marrow stem cells differentiating into non-hematopoietic cells

Early studies indicated that non-hematopoietic marrow “stromal” cells constituted a “hematopoietic inductive microenvironment (HIM) [23]. The spatial distribution of colony -forming unit spleen (CFU-s) and colony-forming unit culture (CFU-c) in normal murine femur showed localization near the endosteum [24]. A niche hypothesis for stem cells was proposed by Schofield [25]. In these studies, approximately half of the CFU-s colonies were on the spleen surface and most of these were erythroid. Granulocytic colonies were detected in sub capsular sheets or along the trabeculae and megakaryocyte colonies were usually sub-capsular [26–28].

Studies on in vitro culture stromal support for either predominantly myeloid cells and stem cells (Dexter cultures) [29] or lymphoid cells (Whitlock-Witte cultures) [30] gave further support for the importance of specific types of non-hematopoietic stromal cells in directing and supporting different hematopoietic lineage choices. The genetic murine models SL/SL^d^ [31] and W/Wv [32] showed specific and separate molecular support for either stromal cells or hematopoietic stem cells, respectively. Subsequent studies utilizing highly purified hematopoietic stem cells focused initially on osteoblasts [33, 34] and endothelial cells [35, 36] as putative niche cells. Further work has indicated that key events may occur around sinusoidal perivascular niches which may involve endothelial cells, pericytes, CXCL abundant reticular cells, sinusoidal-megakaryocyte cells, nestin+ cells, osteoblasts, osteoclasts, T cells, mesenchymal stem cells, macrophages, non-myelinating Schwann cells, and preadipocytic fibroblasts [37–39]. A large number of putative regulators from the niche have also been described, including CXCL12 [40], E-selectin [41], VEGFR 2 [42], N-cadherin [43], osteopontin [44], and membrane bound steel factor [45]. While these entities have various effects on hematopoietic stem cells, their true role as niche maintenance factors is uncertain, except for that of membrane bound SCF. Extensive studies in the SL/SL^d^ mouse model have shown a critical role for this factor in microenvironmental support of hematopoiesis. Many of these studies were potentially confounded by the difficulty in interpreting cellular positional studies and possibly by the utilization of purified stem cells, which are not representative of the total marrow renewing stem cell population.

Perhaps of greatest significance to the universal stem cell concept are the studies on stem cell plasticity, which showed that hematopoietic marrow cells could differentiate into non-hematopoietic cells specific to the tissue in which they engrafted. A large series of publications demonstrated the capacity of marrow cells to give rise to non-hematopoietic cells after in vivo engraftment into irradiated mice [46–53]. This latter was dismissed by many in the field but appears in fact to be real [46]. Work on different marrow cell types contributing to nonhematopoietic cells in lungs of mice subjected to various injuries including radiation [54–58], elastase [59], monocrotaline [60], and bleomycin [61–63] treatments has been impressive. Various transgenic mouse models have also shown marrow cell derived production of lung cells [64–66] and this has also been observed in parabiotic and new born mice [67, 68].

An important contribution was the work of Kraus and colleagues [69]. They harvested marrow from male B6D2/F1 mice These cells were elutriated, and the elutriated male cells were labeled with PKH26, injected into lethally irradiated female mice and fluorescent cells collected from marrow 2 days later. These fluorescent cells were injected as single cells into lethally irradiated female mice. Engraftment, 11 months later, was seen in a variety of tissues but most impressively in lung, where in one mouse over 20% of lung cells were derived from the engrafted single cell. These cells marked as epithelial cells. The key marrow cells generating epithelial lung cells were subsequently shown to be similar to the small embryonic-like stem cells [70] as described by Kucia and colleagues [71]. Twenty-three separate studies have confirmed these single cell results from early marrow cells, many being typical purified hematopoietic stem cells. The one study claiming not to confirm them was quietly, retracted [72].

Our own studies in marrow to lung conversions provides strong support for our Universal Stem Cell model. We initially showed that GFP + marrow cells engrafted in the lungs of mice and produced GFP + epithelial lung cells expressing pulmonary specific mRNA [54]. These conversion phenomena were increased with increasing doses of recipient radiation, and administration of granulocyte colony-stimulating factor to engrafted mice. Lineage negative, c-kit and Sca-1 positive cells showed these conversions, while marrow cells negative for these markers did not. Additional work demonstrated that Lineage-Sca-1+ cells induced to transit cell cycle with cytokines showed marked changes in expression of some genes and not others at different points in cycle [21, 22]. In addition, we showed that marrow cells in culture at G1/S showed marked increases in 3 h homing to marrow and in conversion to epithelial lung cells 6 weeks to 2 months after cell infusion to lethally irradiated mice [73]. This is relatively direct evidence of cycle related stem cell plasticity involving the lung. Cycle related induction of pulmonary mRNA was demonstrated in experiments in which Lineage-Sca-1 positive marrow cells at specific cell cycle phases were co-cultured with normal or irradiated lung cells, conditioned media or extracellular vesicles from these lungs and then analyzed for lung specific mRNA. Increased induction of lung-specific mRNA was seen in G0/G1 Lineage-Sca-1+ cells exposed to irradiated lung and lung vesicles and in G1/S cells when exposed to normal lung or their vesicles [74]. Thus, both the cell cycle status of the marrow stem cells and the nature of the interacting tissue (irradiated or not) determined the extent of pulmonary genotype change seen in marrow stem cells. We also introduced the beginning of the concept that marrow stem cell conversion to producing lung cells could be mediated by lung derived extracellular vesicles entering the marrow stem cells and changing them to lung stem cells (for mechanisms see below) [75, 76]. All together, these observations fit nicely with the concept that marrow stem cells at a certain point in cell cycle can interact with tissue in a particular functional state to produce marrow stem cell derived pulmonary cells, and that the plastic changes may be mediated by lung vesicles interacting with the marrow cells.

**Fig. 3:**
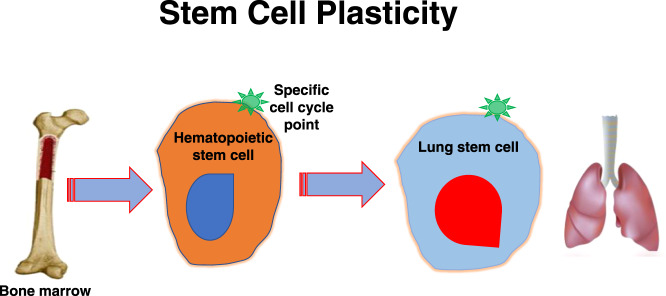
Stem cell plasticity. Orange circle is a standard hematopoietic stem cell at a specific point in cell cycle which favors pulmonary cell differentiation. When this cell is exposed to the lung microenvironment or to vesicles derived from the lung it preferentially differentiates into pulmonary cells. This preferential differentiation occurs with stem cells at different cycle points in most tissues in the mammalian body.

Of further significance to our proposed model are studies indicating that neural tissue cells on engraftment could give rise to hematopoietic marrow-based cells [77], although this was not confirmed by other studies [78]. Overall, it would appear that there may be multiple inductive microenvironments, which may be cellular or tissue specific. Thus, one can envision a putative universal stem cell transiting different organs in vivo and, at an appropriate point in stem cell cycle, giving rise to tissue-specific stem cells (Fig. 3).

An overview of the microenvironment field is presented in Table 2.

**Table 2:** Microenvironment and stem cell plasticity.

1. SL/SL^d^ – Genetic model of stromal insufficiency
2. Physical location of hematopoiesis
3. CFU-s Dexter and Whitlock-Witte stromal cultures
4. Plasticity: Engrafted marrow stem cells giving rise to non-hematopoietic cells in different organs—lung, heart, brain, etc.

## Heterogeneity of stem cells

**Fig. 4:**
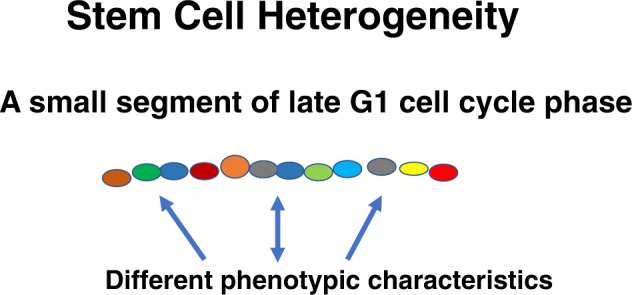
Stem cell heterogeneity. This shows different points in cell cycle with different “stem” cell differentiation characteristics. For instance, a cell at green may favor differentiation into neural cells while a cell at blue favors differentiation into heart cells. A cell at red may be a typical hematopoietic stem cell favoring differentiation into different hematopoietic cell types. These sites of favored differentiation may also represent different previously described stem cells. The time intervals here are unknown but may be quite small.

If the stem cell is cycling that means its phenotype is continually changing. Thus, a different phenotype will be seen at relatively short time intervals of cycle passage. This would protect this critical population from all-or-none disastrous toxicity. This suggests the existence of a stem cell calculous in which the cycle related changes in phenotype represent the derivatives, while the ultimate outcome is the integral. Multiple studies on the heterogeneity of different stem cell classes fit this model [79–85]. Our own work indicated that even highly purified LRH stem cells isolated at different points in cytokine induced cell cycle transit showed almost total heterogeneity [86], although populations of these cells did show overall patterns of differentiation. This is analogous to previous seminal work by Till, McCulloch and Siminovitch on the spleen colony forming unit [87]. A quote from their work follows: “An analogy with the decay of radioactive nuclides may be helpful in this regard. If one studies a large number of radioactive atoms, one sees a very regular pattern of decay, following an exponential law. However, if one studies individual atoms, they are found to decay in an unpredictable fashion, at random. It appears possible that our studies of the progeny of single cells display the random feature of hemopoietic function, while a study of large populations of cells reveals the orderly behavior of the whole system. From this point of view, it is the population as a whole that is regulated rather than individual cells, and it is suggested that control mechanisms act by varying the “birth” and “death” probabilities.” This immediately raises the question of what happened to the stem cell assay Colony-forming Unit Spleen. It was dismissed because of lack of correlation with studies on purified stem cells, but given our data on purified stem cells, perhaps the significance of CFU-s as a stem cell assay should be reconsidered. In any case the high degree of heterogeneity, of even cell cycle synchronized stem cells, is consistent with a continually changing phenotype with cycle passage (Fig. 4). Most recently emphasis has been placed on single cell RNA stem cell research. scRNA in different cell populations shows heterogeneity and application of this to murine hematopoietic stem cells has also shown heterogeneity [88].One can assume that even these purified cells are showing small cell cycle progressions to explain the observed heterogeneity.

## What about other cells?

The hematopoietic field has many other candidate stem/progenitor cells. CFU-s was subdivided into assays in which colonies were counted at earlier or later times after cell infusion; colonies at latter times possibly representing more primitive stem cells. In vitro clonal growth of colonies in semi-solid media with different mature cell types occupied many investigators over a number of years. Work by Bradley and Metcalf [89] and Pluznik and Sachs [90] first described granulocyte macrophage colonies and this was followed by characterizations of cells giving rise to colonies with virtually all differentiated hematopoietic cell types; erythroid [91], megakaryocyte [92], mast cell [93], eosinophil [94], T cell [95], and B cell [96], in varying combinations. Subsequently, blast forming units and progenitors with multilineage potentials [97, 98] were also described. Overall, these hematopoietic colonies varied from single lineage to a variety of multilineage cell types, the multilineage colonies in general needing multiple cytokines to grow, while the single lineage might respond to single cytokines. In general, these cell types were fitted into a hierarchical model, although their isolation for study was not clear cut. We speculate that the more primitive progenitors may be on the cycle dependent continuum. while the cells which have been terminally committed are no longer on the continuum. Other cell classes may or may not be on the continuum. A number of non-hematopoietic stem cells have been isolated from marrow; these include mesenchymal stem cells [99], multipotent adult progenitor cells [100], marrow-isolated adult multilineage inducible cells [101] and unrestricted somatic stem cells found in cord blood [102]. The small embryonic-like stem cell, as noted above, is a candidate cell with capacity to give rise to different cell types [103]. This is a small cell, characterized as CD133 + CXCR4 + CD34 + SSEA-4+ in humans and Sca-1 + CXCR4 + SSEA-1+ Lineage− CD45 − in mice. These very primitive cells may be a precursor of all the others.

There have also been reports of stem/progenitor cells specific to different tissues. These include gastrointestinal stem cells [104], epidermal stem cells [105], hepatic oval cells [106] and neural stem cells (NSCs) [107–109]. Work by Sun et al. [110] suggests quite a different role for differentiated progenitors. Using transposon-tagging for clonally marked cells, their data indicated that long-lived progenitors are the main drivers of steady-state hematopoiesis during most of adulthood. This is of basic interest in the stem cell/progenitor field, but is also consistent with our evolving universal stem cell model. In a similar alternate fashion, the “immortal strand” hypothesis suggests that as the stem cell divides, it selectively retains those sister chromatids containing the older daughter template DNA strands [111]. This has been quite controversial [112] but recent evidence in support of this hypothesis was detected in neurosphere cultures, immortalized mouse tumor cells and muscle stem cells [113–115]. This could also be consistent with the universal stem cell concept.

We hypothesize that the above-described progenitor/stem cells may all exist on a cell cycle related continuum. Admittedly, some cells will have irreversibly differentiated; in the erythroid system one can envision that the BFU-e might be on the continuum cycle, while the more differentiated CFU-e has been lost to terminal differentiation. Other fates, of course, are not ruled out.

Embryonic Stem Cells [116] and Induced Pluripotent Stem Cells [117], while of great interest, have been induced artificially, so their cycle related differentiation potential and possible position on a continuum are not considered in our schema of in vivo stem cell biology.

## What about mechanisms?

This work presents a view of the cellular mechanisms in stem cell biology. Although there has been extensive work on molecular mechanisms involving purified stem cells, we feel that this applies to a dormant stem cell at a particular point in cell cycle. As to the global stem cell population, detailed molecular studies should be in the future. However, we have begun to understand the possible direct mechanism of conversion from one cell type to another. Work on the capacity of tissue derived extracellular vesicles to alter the phenotype of marrow cells has indicated that a mechanism for alteration of phenotype may be the transfer of tissue transcription regulators to the engrafted marrow cells. We previously have shown that extracellular vesicles from murine lung cells are internalized by cells of the marrow giving persistent pulmonary epithelial cell gene and protein expression in vitro and, after transplantation into lethally irradiated mice, an increase in the number of bone marrow-derived pulmonary epithelial cells in the lungs of transplant recipients [54, 73, 74, 118]. Work utilizing rat/mouse extracellular vesicles and determining the species of induced surfactant mRNA in co cultured marrow stem cells indicated that the immediate genetic change was due to transfer from lung cells by extracellular vesicles of both mRNA and a transcriptional modulator, but that long-term stable cellular phenotype changes were due to transfer of translational modulators inducing stable epigenetic changes [76, 118]. Thus, as to the underlying cellular mechanisms of marrow to lung conversions one can envision a marrow “stem cell” at a “lung-prone” point in cell cycle encountering extracellular vesicles from lung tissue, most probably by residence in the tissue, leading to stable epigenetic changes within the marrow stem cell which convert it to a lung stem cell, i.e.,it is a universal stem cell.

## The universal stem cell regulatory model

Present hierarchical stem cell models are conceptually attractive and fit most conventional thinking. Unfortunately, this created the basis for a grand heuristic mistake. As defined by Wikipedia “a heuristic or heuristic technique is any approach to problem solving or self-discovery that employs a practical method that is not guaranteed to be optimal, perfect, or rational, but is nevertheless sufficient for reaching an immediate, short-term goal or approximation”. This was the basis for the conventional hierarchical models for hematopoietic stem cell biology. Representative and availability heuristics might apply here. Much data supported the purification approach: the obviousness of the hierarchical stem cell model with differentiation to end cells, thus, diminishing pursuit of alternative possibilities. The overwhelming concept was that the long-term repopulating hematopoietic stem cell was quiescent, stable and led to a hierarchical system of differentiation into progenitors, followed by end functional hematopoietic cells. This left unexplained the almost total heterogeneity of any stem cell class, even in a synchronized cell cycle transit, and the abundant literature of marrow stem cell classes differentiating into non-hematopoietic cells in a variety of organs and the data of Sun et al. [110] that “long-lived progenitors are the main drivers of steady-state hematopoiesis during most of adulthood”. Early data suggested that caution should be exercised in propositioning hierarchical models for hematopoietic stem cell biology.

From an editorial in Experimental Hematology termed “The Blueness of Stem Cells” [119]; Then Dr. Ogawa came along and messed everything up. He described a bewildering array of different colony types, with from one to five lineages arising from single cells [98, 120–123]. “These were particularly elegant experiments and derived important new insights including showing that within one cell cycle transit totally different lineages may be pursued by two daughter cells from a blast colony. These data were strong evidence against a hierarchical model of hematopoiesis”.

**Fig. 5:**
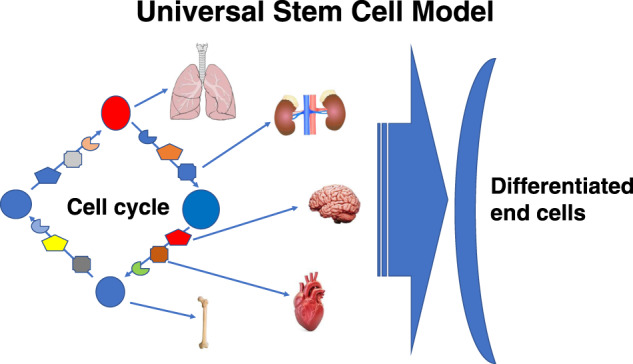
The universal stem cell model. The square of colored entities represents marrow stem cells transiting cell cycle. At certain points in cell cycle, they will have a propensity to differentiate into different cell types in the presence of specific tissues or issues from these tissues (extracellular vesicles). They then can act as lung, kidney, brain, heart, or marrow stem cells as illustrated in the figure.

To restate our model, we propose that there is a universal stem cell which includes the hematopoietic LT-HSC but also multiple other non-hematopoietic stem/progenitor cells in a cycle related stem/progenitor cell continuum. The differentiation of this stem cell then depends upon cell cycle related changes in differentiation potential (illustrated by expression of B220 and Gr-1 and the megakaryocyte hotspot data) and its tissue residence which, via extracellular vesicle modulation, can convert the cell to a non-hematopoietic tissue specific stem cell. This model suggests continual phenotypic changes as the stem cell progresses through cell cycle, immediately explaining the constant heterogeneity observed with different stem/progenitor classes. This also suggests a stem cell calculus in which the cell cycle related phenotype changes are the derivatives and the final population related outcome the integral. This model is pictured in Fig. 5.

Previous work in the stem cell field has laid the groundwork for further progress. The studies on the purified LT-HSC have provided critical insights into this cell at one phase of cell cycle. Future progress extends to characterizing the whole stem cell population.

## What about other factors influencing stem cells on a continuum?

Multiple other factors have been shown to affect the fate of various stem/progenitor cells. These presumably will be active on a continuum. The nature of stem progenitor cells is clearly influenced by circadian rhythm [124–127]. Extracellular vesicles from different tissues represent a moveable microenvironment and impact the fate of progenitor/stem cells [128]. Aging of stem cells is a major area of investigation with many inconclusive results [129, 130]. Clearly this needs to be considered for the effects on a stem cell continuum. There are many other variables that can affect the stem cell fate/phenotype in a continuum, including sex, disease status, race, drug therapy, altitude, and the list continues. In evaluating the nature of the universal stem cell these variables must be considered as they likely play a major role in the observed heterogeneity of stem cells.

## What’s next?

A number of important, but experimentally testable, issues remain. Which cells are not in the continuum, are there multiple continuums, when is differentiation irreversible and do these various parameters change with aging? Can cell cycle specified stem cells be useful in therapies where tissue restoration is critical? Are cancer stem cells also on a continuum and could their definition be clinically useful? Is the stem cell continuum localized to stem cells or is it applicable to all or other cell types? What is the impact of multiple other variables on the fate of stem cells in the continuum?

Altogether, the universal stem cell model suggests exciting new approaches to stem cell biology with intriguing therapeutic implications.

A potential flow of experiments in this area follows:

1. Assess the capacity and purity of engraftable multilineage murine marrow stem cells in marrow cells selected from whole marrow by FACS for different individual or combined sets of stem cell antibodies beginning with anti-Sca-1, anti-c-Kit and anti-CD150.
2. Evaluate the effect of antibodies to Sca-1, c-Kit and CD150 on unseparated marrow cells in the irradiated murine stem cell assays. Also use similar modalities for stem cell separations that utilize dyes for stem cell separations.
3. Take purified lineage negative rhodamine low Hoechst low murine male marrow cells stimulated with steel factor, TPO and FLT3 ligand to synchronously progress through cell cycle and determine their genomic and proteomic profiles at different times of cycle progression. This is an excellent model for synchronous progression through cell cycle of stem cells even though the LRH cell is not indicative of the phenotype of the whole marrow stem cell population. We have previously utilized this model to demonstrate the megakaryocyte and granulocyte differentiation hot spots and shown the tight cell cycle synchrony of these cells when cytokine stimulated.
4. Take B220 or GR1 cells isolated. from male, murine marrow and double sort them, then take the 2 h double sorted B220 or GR-1 negative cells and evaluate their genomic/proteomic profiles at different times in steel alone or in Steel, TPO and Flt3 ligand.
5. Take tissue prone points in cycle determined in 3 and 4 above and evaluate their engraftment into different tissues determining engraftment with either Y positive cells or GFP positive cells and tissue specific markers. The host mice for engraftment would be untreated or subjected to various tissue injuries beginning with irradiation.
6. Harvest vesicles from different normal or perturbed tissues and then evaluate their impact on in vitro cellular differentiation of cells isolated at specific “tissue prone” points in cell cycle.

## Supplementary information

checklist

## Data Availability

Data sharing not applicable to this article as no datasets were generated in this review article.
